# A Rare Vascular Cause of Hoarseness

**DOI:** 10.5334/jbsr.2268

**Published:** 2020-12-01

**Authors:** Florence Calvaer, Pascal Marchetti, Nicolas Brassart

**Affiliations:** 1 CHU Liège, BE; 2 CHU Mons Ambroise Paré, BE

**Keywords:** Ortner syndrome, cardiovocal syndrome, hoarseness, vocal cord paralysis, recurrent laryngeal nerve

## Abstract

**Teaching point:** Hoarseness is a common condition that can be the initial symptom of cardiovascular disorder.

## Case

**Figure 1:**
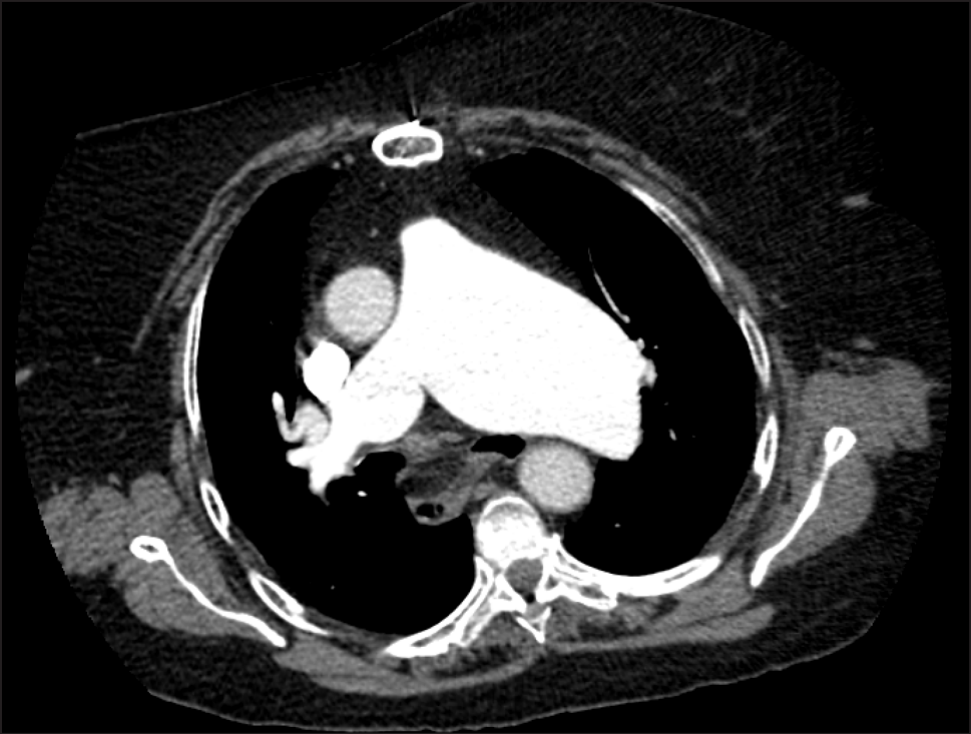


**Figure 2:**
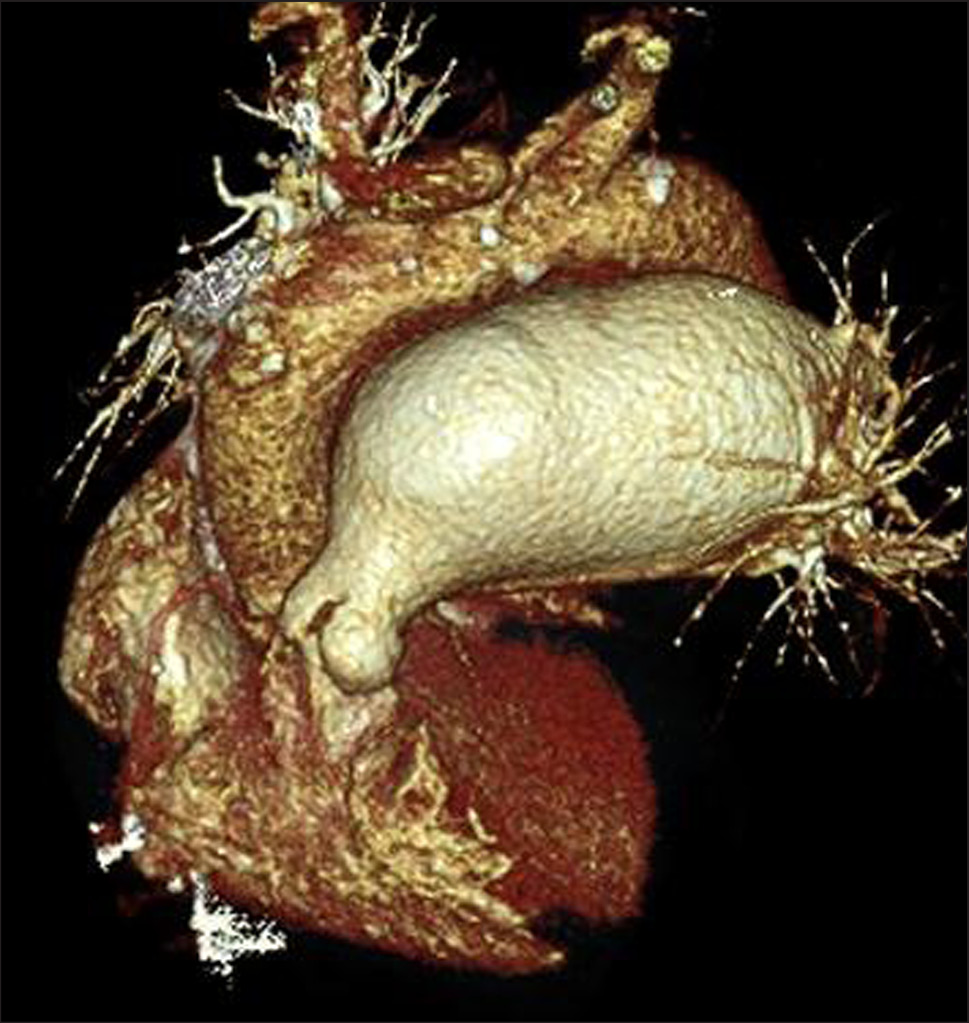


**Figure 3:**
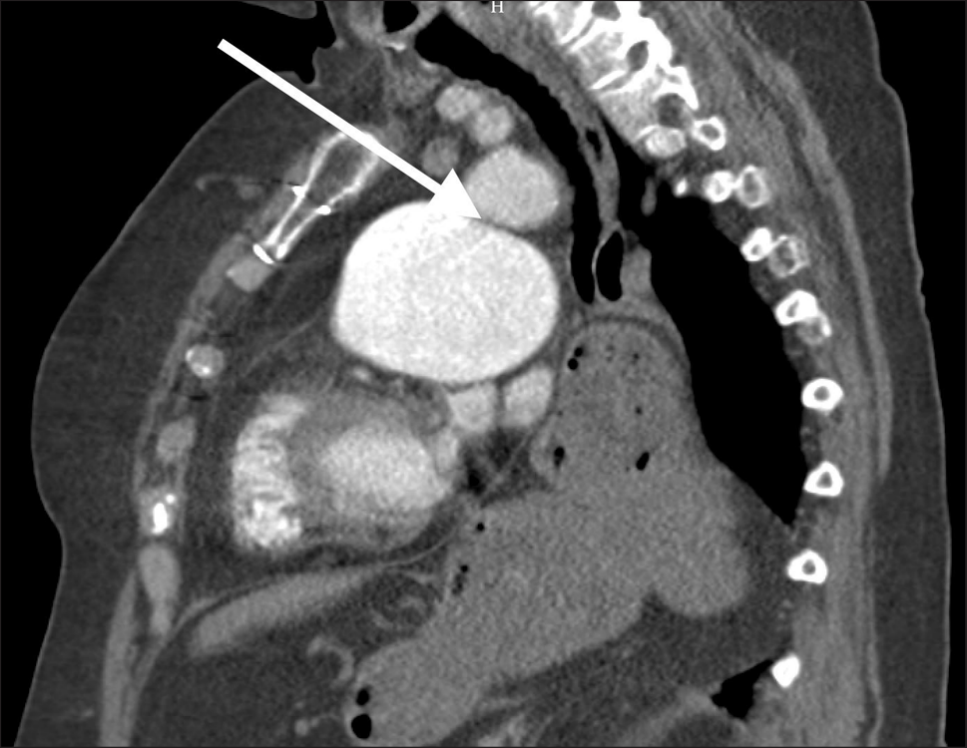


A 76-year-old women who quitted smoking ten years earlier presented with recent-onset hoarseness of the voice. She had history of pulmonary commissurotomy. Fiberoptic laryngoscopy revealed paramedian left vocal cord paralysis. Chest computed tomography showed hiatus hernia and left pulmonary artery aneurysm (largest diameter 66 mm) (Figures 1 and 2) with narrowed aortopulmonary window (Figure 3, arrow).

## Comment

The recurrent laryngeal nerves originate from the vagus nerves at different levels. The right recurrent laryngeal nerve exits anterior to the right subclavian artery and runs underneath and behind it. On the left side, the recurrent laryngeal nerve leaves the vagus nerve on the anterior surface of the aortic arch, running inferiorly around it through the aortopulmonary window posterior to the ligamentum arteriosum. Then they both rise up between the trachea and esophagus to reach larynx and innervate the intrinsic laryngeal muscles, except the cricothyroid muscle. Because of its longer course in mediastinum, the left recurrent laryngeal nerve is more vulnerable [1]. This nerve can be compressed or stretched at any level of its course and sometimes damage happens as it passes through the aortopulmonary window due to a narrow space between the aorta and pulmonary artery.

There are various mediastinal causes for vocal cord paralysis including surgical or iatrogenic injuries, traumatic lesions, inflammatory or infectious diseases, amyloidosis, tumours, and cardiovascular diseases. Ortner syndrome (or cardiovocal syndrome) refers to hoarseness resulting from left recurrent laryngeal nerve paralysis caused by cardiovascular disease [1]. It was first described in 1897 by Norbert Ortner in patients with severe mitral stenosis.

Pulmonary artery aneurysms are rare, predominantly involving main pulmonary arteries, and may be idiopathic or result from congenital (heart defects, connective tissue abnormalities) or acquired (infections, vasculitis, pulmonary arterial hypertension, chronic pulmonary embolism, neoplasms, iatrogenic lesions) causes. It has been reported that early pulmonary valve commissurotomy may induce pulmonary artery aneurysm development due to eccentric right ventricular outflow jet.

This case emphasizes the need to investigate neck and chest when looking for a causative lesion to hoarseness.
